# Single-Dose Toxicity Study on ML171, a Selective NOX1 Inhibitor, in Mice

**DOI:** 10.1155/2021/5515478

**Published:** 2021-05-30

**Authors:** Se-Hyun Oh, Ji-Sun Ahn, Eun-Joo Oh, You-Jin Kim, Ju-Min Yook, Jeong-Hoon Lim, Hee-Yeon Jung, Ji-Young Choi, Chan-Duck Kim, Sun-Hee Park, Yong-Lim Kim, Jang-Hee Cho

**Affiliations:** ^1^Division of Nephrology, Department of Internal Medicine, School of Medicine, Kyungpook National University, Kyungpook National University Hospital, Daegu 41944, Republic of Korea; ^2^Cell and Matrix Research Institute, Kyungpook National University, Daegu 41944, Republic of Korea; ^3^Department of Internal Medicine, School of Medicine, Kyungpook National University, Daegu 41944, Republic of Korea

## Abstract

**Background:**

ML171 is a potent nicotinamide adenine dinucleotide phosphate oxidase (NOX) inhibitor with isoform selectivity only for NOX1. This study is aimed at investigating the safety of ML171 after a single intraperitoneal (IP) injection in mice.

**Methods:**

The toxicity of a single dose of ML171 was evaluated in 6-week-old Institute of Cancer Research (ICR) mice in a good laboratory practice (GLP) laboratory. Twenty-five mice of each sex were assigned to five groups: negative control, vehicle control, and 125, 250, and 500 mg/kg of ML171. All mice were acclimatized for one week before beginning the study. Mice received an IP injection of ML171 or vehicle. The general condition and mortality of the animals were observed. The mice were sacrificed to evaluate histopathology 14 days after the administration of ML171 or vehicle.

**Results:**

Bodyweights were not significantly different in any group. Three males and one female died due to ML171 administration in the 500 mg/kg dose group. Autopsies of the surviving mice did not reveal any significant abnormalities after the injection of 125 mg/kg of ML171. However, the anterior lobe edge of the liver was thickened and adhesions between the liver and adjacent organs were observed in mice treated with 250 or 500 mg/kg of ML171. In addition, hypertrophy of centrilobular hepatocytes and inflammatory cell infiltration were observed after injection of 250 and 500 mg/kg of ML171.

**Conclusion:**

Our results indicate that the lethal IP injection dose of ML171 is 500 mg/kg for both males and females. Mortality were not observed for lower doses of ML171. The safe dose of single IP ML171 in ICR mice was 250 mg/kg or less. Further studies are needed to confirm the safety of ML171 in the human body.

## 1. Introduction

The nicotinamide adenine dinucleotide phosphate oxidase (NOX) family catalyzes reactive oxygen species (ROS) [1, 2]. NOX generates ROS, which play a role in a growing number of diseases, including cancer, atherosclerosis, hypertension, neurological disorders, and inflammation [3–5]. Among the NOX inhibitors, ML171 is a potent, selective inhibitor of NOX1, with an IC50 of 129–156 nM in cell-based assays. ML171 blocks NOX1-dependent ROS generation, with only marginal inhibitory effects on other cellular ROS-producing enzymes and receptors, including the other NOX isoforms [6].

ML171 is effective in inhibiting oxidative stress [7, 8]. The antioxidant effects of ML171 have been studied in a variety of physiological and pathological processes, including the development and effects of severe hypertension, metastasis of hepatocellular carcinoma, and glial activation with crosstalk control [9–11]. Recently, we confirmed the renoprotective effect of ML171 in an ischemia-reperfusion injury (IRI) mouse model. ML171 attenuated kidney IRI via inhibition of ROS-mediated Extracellular Signal-Regulated Kinase (ERK) signaling and reduced oxidative stress-induced apoptosis in renal tubule cells [12]. These results suggest that ML171 could be used as a therapeutic agent for oxidative injury in many different organs.

In a preclinical study, we treated animals with a 60 mg/kg intraperitoneal (IP) injection of ML171. The IP injection was well tolerated in all the mice without any adverse events and histological changes [12]. However, the experiments were not performed in a good laboratory practice (GLP) setting. A more objective and systematic verification of ML171 safety was needed before human clinical trials. This study is aimed at investigating the safety of ML171 after a single IP injection in mice in compliance with GLP and at determining the approximate lethal dose of ML171.

## 2. Materials and Methods

### 2.1. Study Approval

Animal experiments were conducted by Biotoxtech (Cheongwon, Korea, Biotoxtech study number B20052) in accordance with the guidelines of the Food and Drug Administration (KFDA, 2017) and the Principles of Good Laboratory Practices of the Organization for Economic Cooperation and Development (OECD, 1998) [13]. All experiments and histology analysis were performed according to that “ICH Harmonised Tripartite Guideline, M3 (R2), Guidance on Nonclinical Safety Studies for the Conduct of Human Clinical Trials and Marketing Authorization for Pharmaceuticals” [14]. Approval for this study was granted from the Institutional Animal Ethics Committee of Biotoxtech (200133).

### 2.2. Preparation of ML171 and Vehicle Control Solutions

The ML171 (2-acetylphenothiazine; HY-12805, MedChemExpress, U.S.A.) was weighed and placed in a preparation bottle. The drug was dissolved in a sterile solution of 10% DMSO (K50270931, Merck, Germany), 40% PEG300 (BCCB1565, Sigma-Aldrich, Co., USA), and 5% Tween-80 (BCBV8843, Sigma-Aldrich, Co., USA) in physiological saline (19092, JW Pharmaceutical Co., Ltd., Korea). The vehicle control material was 10% DMSO, 40% PEG300, and 5% Tween-80 in physiological saline. The ML171 and vehicle solution were prepared just before administration.

### 2.3. Single-Dose IP Toxicity Test of ML171 in Mice

Six-week-old female and male Institute of Cancer Research (ICR) mice were used for the study (Orient Bio Inc., Seongnam, Korea). After a one-week acclimatization period, the bodyweight and general health were checked to confirm no abnormalities in all animals. Male and female (25 each) mice with similar bodyweights were selected and divided into five groups as follows: G1/0, negative control; G2/0, vehicle control; G3/125, 125 mg/kg of ML171; G4, 250 mg/kg of ML171; and G5, 500 mg/kg of ML171. Animals were randomly assigned to groups, so the average weights were similar between groups.

ML171 was administered by an IP injection to evaluate abdominal cavity exposure to the drug. The injection volume for each individual was calculated based on the bodyweight before administering the injection. The injection volume was 20 ml/kg, and the doses were 125 mg/kg, 250 mg/kg, and 500 mg/kg. The dosage was based on preliminary data, and the dosages were selected in a range in which no mortality was observed in preliminary experiments. The same amount of physiological saline or vehicle (10% DMSO, 40% PEG300, and 5% Tween-80) was administered to the control and vehicle control groups, respectively.

### 2.4. General Observation and Histopathological Analysis of Mice

General health, including the type of toxic symptoms, time of onset, and recovery time, was evaluated at 1, 2, 4, and 6 hours after drug or vehicle administration. General health was examined once daily for 14 days after the first day of drug administration. Deaths were recorded. Bodyweight was measured before drug administration and 4, 8, and 15 days after administration (autopsy day). If a dead animal was observed during the observation period, the bodyweight was measured on the day of death and the animal was autopsied within 24 hours. All surviving mice were sacrificed at 15 days, and histological examinations were performed. Gross findings were recorded during the autopsy, and a histopathological examination with hematoxylin and eosin (H&E) staining of the liver was performed [15].

### 2.5. Statistical Analysis

Data are expressed as the mean ± standard deviation for the bodyweight and numbers for the number of mice. Bartlett's test was performed to test for an equal variance of the bodyweight. In the case of equal variance, significance was confirmed by one-way analysis of variance. Mortality, the incidence of macroscopic or microscopic findings in the liver, was analyzed by a chi-square test. The statistical analysis was performed using Statistical Analysis System (SAS) (version 9.3, SAS Institute Inc., USA). *p* < 0.05 was considered statistically significant.

## 3. Results

### 3.1. Effects of ML171 on Bodyweight

**Figure 1:**
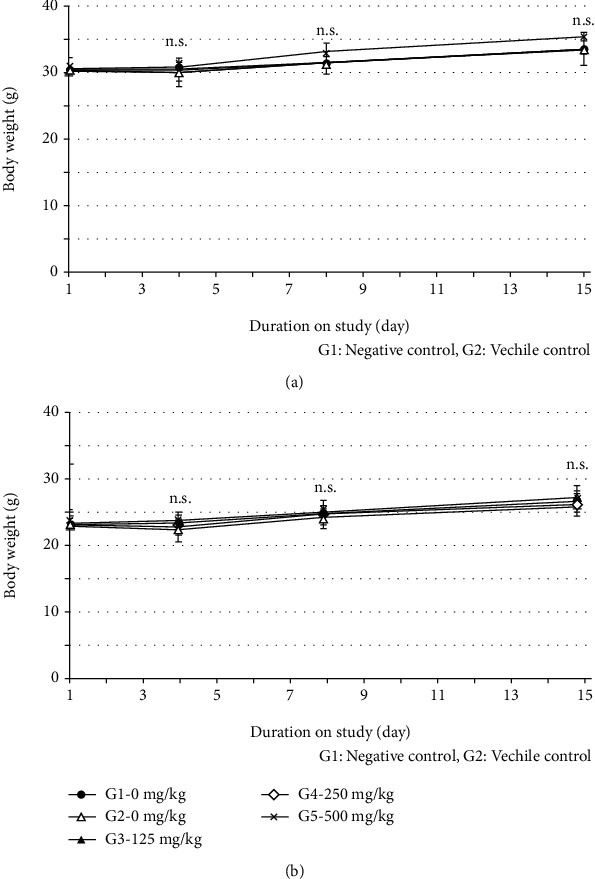
Bodyweights in male (a) and female (b) mice after a single injection of ML171 or vehicle. G1/0 is the untreated control group, and G2/0 is the vehicle-treated group. The G3/125, G4/250, and G5/500 groups were treated with the indicated dose of ML171. The *x*-axis indicates days after injection. Data represent the mean ± SD, and there is no significant difference in one-way analysis of variance (*p* > 0.05). n.s.: not significant.

Bodyweight changes for male and female mice are summarized in Table 1. The ML171 groups, including G3/125, G4/250, and G5/500, showed comparable bodyweight changes compared to the G1/0 and G2/0 groups (Figure 1). There were no significant differences (*p* > 0.05) in the bodyweights among male or female groups throughout the study.

### 3.2. Effects of ML171 on General Health and Mortality

During the observation period, no abnormalities in general health were observed in either male or female mice in the G1/0, G2/0, G3/125, and G4/250 groups. In the G3/125 group, one male mouse showed yellow-colored mucous stool 0.5, 1, and 4 hours after administration. Two female mice in the G3/125 group also showed mucous stools 0.5 hours after administration.

Three males in the G5/500 group exhibited decreased locomotor activity and fecal volume with tremor two days after administration. All three mice died on the third day after injection and were found prone or lying on their side (Table 2). Two females in the G5/500 group also showed a decrease in fecal volume and tremor on the second day after drug administration. Decreased spontaneous movement with irregular respiration was observed on the third day in one of the mice, and the mouse died on the fourth day after drug injection. During the observation period, individual clinical signs of all mice are detailed in Supplementary Table 1.

### 3.3. Effects of ML171 on Autopsy Findings

Autopsies of the dead mice showed no gross abnormalities. In the surviving cases, no gross abnormalities were observed in either male or female mice from the G1/0, G2/0, and G3/125 groups. However, thickening in the anterior lobular edge of the liver was observed in mice from the G4/250 and G5/500 groups. In addition, liver adhesions with the adjacent stomach, kidney, and diaphragm were observed in the G4/250 and G5/500 groups (Table 3). There were no significant differences (*p* > 0.05) in the frequency of macroscopic abnormalities among groups.

### 3.4. Effects of ML171 on the Histology of Injection Sites

**Figure 2:**
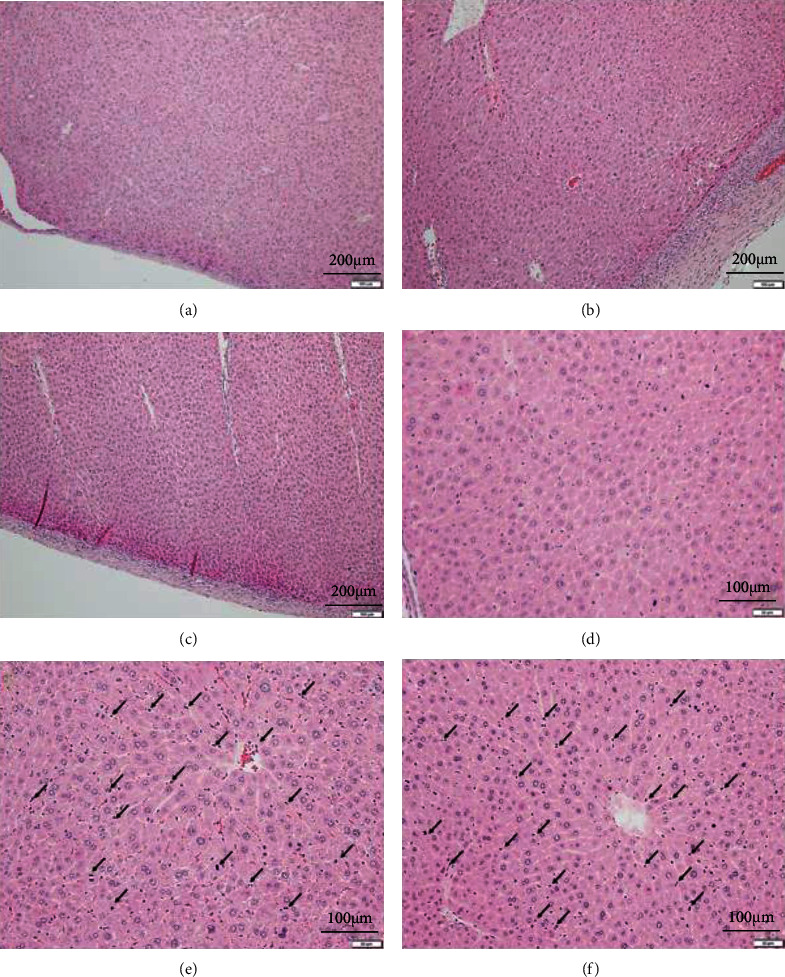
Histology of the liver. G1/0 (a, d) is vehicle control groups. Mild fibrosis of the membrane and inflammatory cell infiltration were observed in the G4/250 (b) and G5/500 (c) groups. Hepatocytes were hypertrophic in centrilobular areas and increased mitosis occurred in the G4/250 (e) and G5/500 (f) groups.

Histology of the liver revealed mild to moderate hypertrophy of centrilobular hepatocytes in three males and one female in the G4/250 group and all males and two females in the G5/500 group. This finding correlated with the lobe thickening observed in the autopsy. Hepatocyte mitosis increased in two males and one female in the G4/250 group and all males and three females in the G5/500 group. In addition, mild fibrosis of the membrane and inflammatory cell infiltration were observed in all mice from the G4/250 and G5/500 groups. The frequency of microscopic abnormalities did not differ significantly (*p* > 0.05) among groups (Table 4). The fibrosis and inflammation were associated with the liver adhesions to adjacent organs and tissues in the abdominal cavity. Representative images and the frequency of the findings are shown in Figure 2 and Table 4, respectively.

## 4. Discussion

We investigated the safety of a single IP injection of ML171 in a GLP laboratory. Bodyweight was not affected by the administration of ML171 at any of the doses. However, four mice (40%) treated with 500 mg/kg of ML171 died. The autopsies of the surviving cases did not reveal any significant abnormalities in the control and G3/125 groups. However, the anterior lobe edge of the liver was thickened and adhesions of the liver with adjacent organs were observed in the G4/250 and G5/500 groups. In addition, hypertrophy of centrilobular hepatocytes and inflammatory cell infiltration were observed in the G4/250 and G5/500 groups. These results suggest that the safe dose of single IP ML171 in ICR mice was 250 mg/kg or less.

The single-dose toxicity test study determines toxic phenomena occurring within a short time after a single test substance administration. This test confirms a safe single dose that does not cause adverse events or life-threatening effects to the animal. The present test was conducted to evaluate IP injection toxicity and obtain a lethal dose of ML171. Forty percent of ICR mice were injected with 500 mg/kg of ML171 died, whereas mortality was not observed in the lower dose groups. Injection of 250 mg/kg of ML171 caused macroscopic changes, such as thickening of the lobular edge of the liver and adhesion of the liver to surrounding organs, which corresponded to microscopic abnormalities. A dose higher than 250 mg/kg of ML171 might induce more toxicity in mice; therefore, 250 mg/kg is the highest dose of ML171 to expect efficacy without significant adverse effects such as mortality.

Among the seven NOX isoforms [16], NOX1-dependent ROS production contributes to cellular growth signaling, angiogenesis, motility, and endothelial function [17–19]. NOX1 is a therapeutic target for IRI of multiple organs, including the heart, lung, kidney, and retina [12, 20–22]. ML171, a selective NOX1 inhibitor, has been studied in various animal models [11, 23, 24]. The route of ML171 administration was a single IP injection, ranging between 20 and 60 mg/kg. The present single-dose toxicity study tested the safety of a single IP administration of ML171 with a range between 125–500 mg/kg. Therefore, we secured the safety evidence for a single IP dosing of ML171. However, further safety toxicity tests, such as long-term recovery tests and repeated dose toxicity tests, are needed to provide more safety data.

Autopsies of mice treated with higher ML171 doses revealed a thickening of the anterior hepatic lobes. The anterior location may have been directly exposed to the injected ML171. The lobe thickening correlated with the histologic finding of hepatocyte hypertrophy. Macroscopic liver adhesions to the surrounding organs were confirmed by fibrosis of the membranes and inflammatory cell infiltration. These changes, along with the increased hepatocyte mitosis, are generally regarded as a repair response after haptic injury and hepatocyte loss [25]. Additional blood tests are required to evaluate the degree of liver damage. However, we did not find any macroscopic and microscopic abnormalities in mice after injection with 125 mg/kg of ML171. Liang et al. reported no other liver pathology after multiple injections of ML171 in mice with hepatocellular carcinoma [10]. In Liang et al.'s study, ML171 was administered intraperitoneally twice weekly for four weeks. Together with the present study, these results demonstrate the long-term safety of ML171 in terms of hepatotoxicity.

Sexual differences in toxic symptoms occur in response to some drugs [26, 27]. Thus, we evaluated both male and female mice in the present study. The bodyweight consistently increased over time in both male and female mice. Adverse symptoms and mortality were not significantly different between the sexes. Furthermore, macroscopic and microscopic findings observed in the G4/250 and G5/500 groups occurred in both males and females. Our results suggest that ML171 toxicities are not associated with sex and the lethal dose is not different between males and females.

There are several limitations to the present study. As described above, recovery tests and repeated dose toxicity tests should be performed to evaluate the long-term effects of ML171. Further studies should include a biochemical analysis of the 250 mg/kg dose, which induced some histologic changes to the liver. The detailed doses between 125 and 250 should be explored to identify the true cutoff for toxic outcomes. If persistent liver damage is observed, an analysis of the underlying mechanism should also be performed. Nevertheless, our research is the first study to demonstrate the safety of a single ML171 injection and the lethal dose for IP injection of ML171 in a mouse model. These data form the basis for future long-term experiments with ML171.

## 5. Conclusions

The results of this study indicate that the lethal IP dose of ML171 is 500 mg/kg for both males and females. Mortality was not observed in doses up to 250 mg/kg of ML171. Thus, the safe dose of single IP ML171 in ICR mice was 250 mg/kg or less. Further studies are needed to confirm the safety of ML171 in the human body.

## Figures and Tables

**Table 1:** Mean bodyweights.

Sex	Group/dose (mg/kg)		Day			
			1	4	8	15
Male	G1/0	Mean	30.3	30.8	31.5	33.5
		SD	0.7	0.7	0.9	1.6
		*N*	5	5	5	5
	G2/0	Mean	0.2	30.1	31.6	33.4
		SD	1.2	2.1	1.8	1.8
		*N*	5	5	5	5
	G3/125	Mean	30.5	30.5	31.4	33.6
		SD	1.7	1.7	1.8	1.9
		*N*	5	5	5	5
	G4/250	Mean	30.4	30.2	31.5	33.4
		SD	1.2	1.4	1	2.2
		*N*	5	5	5	5
	G5/500	Mean	30.4	30.8	33.0	35.4
		SD	1.4	0.2	1.5	0.6
		*N*	5	2	2	2

Female	G1/0	Mean	22.8	22.9	24.3	26.3
		SD	1.8	1.3	2.1	1.1
		*N*	5	5	5	5
	G2/0	Mean	22.9	22.0	23.8	25.8
		SD	0.9	0.3	1.0	1.7
		*N*	5	5	5	5
	G3/125	Mean	22.6	23.3	24.6	26.9
		SD	1.5	1.4	1.9	1.7
		*N*	5	5	5	5
	G4/250	Mean	22.6	22.4	24.2	25.5
		SD	1.4	1.1	1.0	1.2
		*N*	5	5	5	5
	G5/500	Mean	22.9	22.4	24.7	26.2
		SD	1.4	2.2	1.0	1.6
		N	5	4	4	4

G1: negative control; G2: vehicle control. Data represent the mean ± SD, and no significant difference was found in one-way analysis of variance (*p* > 0.05).

**Table 2:** Summary of mortality.

Sex	Group/dose (mg/kg)	No. of animals	Day after treatment															Mortality
			1	2	3	4	5	6	7	8	9	10	11	12	13	14	15	
Male	G1/0	5	^∗^0	0	0	0	0	0	0	0	0	0	0	0	0	0	0	^#^0/5
	G2/0	5	0	0	0	0	0	0	0	0	0	0	0	0	0	0	0	0/5
	G3/125	5	0	0	0	0	0	0	0	0	0	0	0	0	0	0	0	0/5
	G4/250	5	0	0	0	0	0	0	0	0	0	0	0	0	0	0	0	0/5
	G5/500	5	0	0	3	0	0	0	0	0	0	0	0	0	0	0	0	3/5

Female	G1/0	5	0	0	0	0	0	0	0	0	0	0	0	0	0	0	0	0/5
	G2/0	5	0	0	0	0	0	0	0	0	0	0	0	0	0	0	0	0/5
	G3/125	5	0	0	0	0	0	0	0	0	0	0	0	0	0	0	0	0/5
	G4/250	5	0	0	0	0	0	0	0	0	0	0	0	0	0	0	0	0/5
	G5/500	5	0	0	0	1	0	0	0	0	0	0	0	0	0	0	0	1/5

G1: negative control; G2: vehicle control. ^∗^Number of dead mice. ^#^Number of with mortality/total mice.

**(a):** 

Organ/findings	Sex	Male				
	Group/dose (mg/kg)	G1/0	G2/0	G3/125	G4/250	G5/500
		Negative control	Vehicle control	Low dose	Mid dose	High dose
Number examined		5	5	5	5	2
Liver						
Thickening, lobar edge, all lobes		^∗^0	0	0	5	2
*Total number of affected animals*		^#^0/5	0/5	0/5	5/5	2/2
Adhesion, to adjacent organs/tissues (stomach, diaphragm)		^∗^0	0	0	5	2
*Total number of affected animals*		^#^0/5	0/5	0/5	5/5	2/2

**(b):** 

Organ/findings	Sex	Female				
	Group/dose (mg/kg)	G1/0	G2/0	G3/125	G4/250	G5/500
		Negative control	Vehicle control	Low dose	Mid dose	High dose
Number examined		5	5	5	5	4
Liver						
Thickening, lobar edge, all lobes		^∗^0	0	0	5	4
*Total number of affected animals*		^#^0/5	0/5	0/5	5/5	4/5
Adhesion, to adjacent organs/tissues (stomach, diaphragm)		^∗^0	0	0	5	4
*Total number of affected animals*		^#^0/5	0/5	0/5	5/5	4/5

^∗^Number of affected mice on liver. ^#^Number of with affected mice/total mice. Data was no significant difference after using the chi-square test (all *p* > 0.05).

**Table 4:** Incidence and severity of microscopic findings in the liver.

Tissue/findings	Sex	Male		Female	
	Group/dose (mg/kg)	G4/250	G5/500	G4/250	G5/500
		Mid dose	High dose	Mid dose	High dose
Number examined		5	2	5	4
Liver					
Fibrosis, capsular, with inflammatory cell infiltrate					
Minimal		^∗^5	2	5	4
*Total number of affected animals*		^#^5/5	2/2	5/5	4/4
Increased mitosis, hepatocyte					
Minimal		^∗^2	2	1	3
*Total number of affected animals*		^#^2/5	2	1	3
Hypertrophy, hepatocyte, centrilobular					
Minimal		^∗^2	0	1	1
Slight		^∗^1	2	0	1
*Total number of affected animals*		^#^3/5	2/2	1/5	2/4

^∗^Number of affected mice on the liver. ^#^Number of with affected mice/total mice. Data was no significant difference after using the chi-square test (all *p* > 0.05).

## Data Availability

The data that support the findings of this study are available from the corresponding author upon reasonable request.

## Supplementary Materials

Supplementary Materials Supplemental Table 1: individual clinical signs.
