# Supplementary material for: Single-Dose Toxicity Study on ML171, a Selective NOX1 Inhibitor, in Mice
Source: Biomed Res Int. 2021 May 30;2021:5515478. doi: 10.1155/2021/5515478 (PMC8181097; doi:10.1155/2021/5515478)
Supplement: Supplementary Materials — Supplemental Table 1: individual clinical signs. [file 5515478.f1.docx]

**Supplemental Table 1. Individual Clinical Signs**

**Sex : Male**

| **Group / Dose (mg/kg)** | **Animal ID** | **Clinical sign** | **Hours (Day 1) after treatment** | | | | |
| --- | --- | --- | --- | --- | --- | --- | --- |
|  |  |  | **0.5** | **1** | **2** | **4** | **6** |
| **G1/0** | 1 |  | - | - | - | - | - |
|  | 2 |  | - | - | - | - | - |
|  | 3 |  | - | - | - | - | - |
|  | 4 |  | - | - | - | - | - |
|  | 5 |  | - | - | - | - | - |
| **G2/0** | 1 |  | - | - | - | - | - |
|  | 2 |  | - | - | - | - | - |
|  | 3 |  | - | - | - | - | - |
|  | 4 |  | - | - | - | - | - |
|  | 5 |  | - | - | - | - | - |
| **G3/125** | 1 |  | - | - | - | - | - |
|  | 2 |  | - | - | - | - | - |
|  | 3 |  | - | - | - | - | - |
|  | 4 |  | - | - | - | - | - |
|  | 5 | Colored stool (yellow) | **+** | **+** | - | **+** | - |
|  |  | Mucous stool | **+** | **+** | - | **+** | - |
| **G4/250** | 1 |  | - | - | - | - | - |
|  | 2 |  | - | - | - | - | - |
|  | 3 |  | - | - | - | - | - |
|  | 4 |  | - | - | - | - | - |
|  | 5 |  | - | - | - | - | - |
| **G5/500** | 1 |  | - | - | - | - | - |
|  | 2 |  | - | - | - | - | - |
|  | 3 |  | - | - | - | - | - |
|  | 4 |  | - | - | - | - | - |
|  | 5 |  | - | - | - | - | - |

-: No observable abnormality

+: Observable abnormality

G1: Negative control, G2: Vehicle control

| **Group / Dose (mg/kg)** | **Animal ID** | **Clinical sign** | **Day of after treatment** | | | | | | | | | | | | | |
| --- | --- | --- | --- | --- | --- | --- | --- | --- | --- | --- | --- | --- | --- | --- | --- | --- |
|  |  |  | **2** | **3** | **4** | **5** | **6** | **7** | **8** | **9** | **10** | **11** | **12** | **13** | **14** | **15** |
| **G1/0** | 1 |  | - | - | - | - | - | - | - | - | - | - | - | - | - | - |
|  | 2 |  | - | - | - | - | - | - | - | - | - | - | - | - | - | - |
|  | 3 |  | - | - | - | - | - | - | - | - | - | - | - | - | - | - |
|  | 4 |  | - | - | - | - | - | - | - | - | - | - | - | - | - | - |
|  | 5 |  | - | - | - | - | - | - | - | - | - | - | - | - | - | - |
| **G2/0** | 1 |  | - | - | - | - | - | - | - | - | - | - | - | - | - | - |
|  | 2 |  | - | - | - | - | - | - | - | - | - | - | - | - | - | - |
|  | 3 |  | - | - | - | - | - | - | - | - | - | - | - | - | - | - |
|  | 4 |  | - | - | - | - | - | - | - | - | - | - | - | - | - | - |
|  | 5 |  | - | - | - | - | - | - | - | - | - | - | - | - | - | - |
| **G3/125** | 1 |  | - | - | - | - | - | - | - | - | - | - | - | - | - | - |
|  | 2 |  | - | - | - | - | - | - | - | - | - | - | - | - | - | - |
|  | 3 |  | - | - | - | - | - | - | - | - | - | - | - | - | - | - |
|  | 4 |  | - | - | - | - | - | - | - | - | - | - | - | - | - | - |
|  | 5 |  | - | - | - | - | - | - | - | - | - | - | - | - | - | - |
| **G4/250** | 1 |  | - | - | - | - | - | - | - | - | - | - | - | - | - | - |
|  | 2 |  | - | - | - | - | - | - | - | - | - | - | - | - | - | - |
|  | 3 |  | - | - | - | - | - | - | - | - | - | - | - | - | - | - |
|  | 4 |  | - | - | - | - | - | - | - | - | - | - | - | - | - | - |
|  | 5 |  | - | - | - | - | - | - | - | - | - | - | - | - | - | - |
| **G5/500** | 1 |  | - | - | - | - | - | - | - | - | - | - | - | - | - | - |
|  | 2 | Decrease of fecal volume | **+** | - |  |  |  |  |  |  |  |  |  |  |  |  |
|  |  | Tremor | **+** | - |  |  |  |  |  |  |  |  |  |  |  |  |
|  |  | Prone position | - | **+** |  |  |  |  |  |  |  |  |  |  |  |  |
|  |  | Death | - | **+** |  |  |  |  |  |  |  |  |  |  |  |  |
|  | 3 | Decrease of fecal volume | **+** | - |  |  |  |  |  |  |  |  |  |  |  |  |
|  |  | Tremor | **+** | - |  |  |  |  |  |  |  |  |  |  |  |  |
|  |  | Lying on side | - | **+** |  |  |  |  |  |  |  |  |  |  |  |  |
|  |  | Death | - | **+** |  |  |  |  |  |  |  |  |  |  |  |  |
|  | 4 | Decrease in locomotor activity | **+** | - |  |  |  |  |  |  |  |  |  |  |  |  |
|  |  | Decrease of fecal volume | **+** | - |  |  |  |  |  |  |  |  |  |  |  |  |
|  |  | Prone position | - | **+** |  |  |  |  |  |  |  |  |  |  |  |  |
|  |  | Death | - | **+** |  |  |  |  |  |  |  |  |  |  |  |  |
|  | 5 |  | - | - | - | - | - | - | - | - | - | - | - | - | - | - |

-: No observable abnormality

+: Observable abnormality

G1: Negative control, G2: Vehicle control

**Sex : Female**

| **Group / Dose (mg/kg)** | **Animal ID** | **Clinical sign** | **Hours (Day 1) after treatment** | | | | |
| --- | --- | --- | --- | --- | --- | --- | --- |
|  |  |  | **0.5** | **1** | **2** | **4** | **6** |
| **G1/0** | 1 |  | - | - | - | - | - |
|  | 2 |  | - | - | - | - | - |
|  | 3 |  | - | - | - | - | - |
|  | 4 |  | - | - | - | - | - |
|  | 5 |  | - | - | - | - | - |
| **G2/0** | 1 |  | - | - | - | - | - |
|  | 2 |  | - | - | - | - | - |
|  | 3 |  | - | - | - | - | - |
|  | 4 |  | - | - | - | - | - |
|  | 5 |  | - | - | - | - | - |
| **G3/125** | 1 |  | - | - | - | - | - |
|  | 2 | Colored stool (yellow) | **+** | - | - | - | - |
|  |  | Mucous stool | **+** | - | - | - | - |
|  | 3 | Colored stool (yellow) | **+** | - | - | - | - |
|  |  | Mucous stool | **+** | - | - | - | - |
|  | 4 |  | - | - | - | - | - |
|  | 5 |  | - | - | - | - | - |
| **G4/250** | 1 |  | - | - | - | - | - |
|  | 2 |  | - | - | - | - | - |
|  | 3 |  | - | - | - | - | - |
|  | 4 |  | - | - | - | - | - |
|  | 5 |  | - | - | - | - | - |
| **G5/500** | 1 |  | - | - | - | - | - |
|  | 2 |  | - | - | - | - | - |
|  | 3 |  | - | - | - | - | - |
|  | 4 |  | - | - | - | - | - |
|  | 5 |  | - | - | - | - | - |

-: No observable abnormality

+: Observable abnormality

G1: Negative control, G2: Vehicle control

| **Group / Dose (mg/kg)** | **Animal ID** | **Clinical sign** | **Day of after treatment** | | | | | | | | | | | | | |
| --- | --- | --- | --- | --- | --- | --- | --- | --- | --- | --- | --- | --- | --- | --- | --- | --- |
|  |  |  | **2** | **3** | **4** | **5** | **6** | **7** | **8** | **9** | **10** | **11** | **12** | **13** | **14** | **15** |
| **G1/0** | 1 |  | - | - | - | - | - | - | - | - | - | - | - | - | - | - |
|  | 2 |  | - | - | - | - | - | - | - | - | - | - | - | - | - | - |
|  | 3 |  | - | - | - | - | - | - | - | - | - | - | - | - | - | - |
|  | 4 |  | - | - | - | - | - | - | - | - | - | - | - | - | - | - |
|  | 5 |  | - | - | - | - | - | - | - | - | - | - | - | - | - | - |
| **G2/0** | 1 |  | - | - | - | - | - | - | - | - | - | - | - | - | - | - |
|  | 2 |  | - | - | - | - | - | - | - | - | - | - | - | - | - | - |
|  | 3 |  | - | - | - | - | - | - | - | - | - | - | - | - | - | - |
|  | 4 |  | - | - | - | - | - | - | - | - | - | - | - | - | - | - |
|  | 5 |  | - | - | - | - | - | - | - | - | - | - | - | - | - | - |
| **G3/125** | 1 |  | - | - | - | - | - | - | - | - | - | - | - | - | - | - |
|  | 2 |  | - | - | - | - | - | - | - | - | - | - | - | - | - | - |
|  | 3 |  | - | - | - | - | - | - | - | - | - | - | - | - | - | - |
|  | 4 |  | - | - | - | - | - | - | - | - | - | - | - | - | - | - |
|  | 5 |  | - | - | - | - | - | - | - | - | - | - | - | - | - | - |
| **G4/250** | 1 |  | - | - | - | - | - | - | - | - | - | - | - | - | - | - |
|  | 2 |  | - | - | - | - | - | - | - | - | - | - | - | - | - | - |
|  | 3 |  | - | - | - | - | - | - | - | - | - | - | - | - | - | - |
|  | 4 |  | - | - | - | - | - | - | - | - | - | - | - | - | - | - |
|  | 5 |  | - | - | - | - | - | - | - | - | - | - | - | - | - | - |
| **G5/500** | 1 |  | - | - | - | - | - | - | - | - | - | - | - | - | - | - |
|  | 2 |  | - | - | - |  |  |  |  |  |  |  |  |  |  |  |
|  | 3 | Decrease of fecal volume | **+** | - | - |  |  |  |  |  |  |  |  |  |  |  |
|  |  | Tremor | **+** | - | - |  |  |  |  |  |  |  |  |  |  |  |
|  |  | Decrease in locomotor activity | - | **+** | - |  |  |  |  |  |  |  |  |  |  |  |
|  |  | Irregular respiration | - | **+** | - |  |  |  |  |  |  |  |  |  |  |  |
|  |  | No stool | - | **+** | - |  |  |  |  |  |  |  |  |  |  |  |
|  |  | Prone position | - | - | **+** |  |  |  |  |  |  |  |  |  |  |  |
|  |  | Death | - | - | **+** |  |  |  |  |  |  |  |  |  |  |  |
|  | 4 |  | - | - | - | - | - | - | - | - | - | - | - | - | - | - |
|  | 5 | Decrease of fecal volume | **+** | - | - | - | - | - | - | - | - | - | - | - | - | - |

-: No observable abnormality

+: Observable abnormality

G1: Negative control, G2: Vehicle control
